# Identification of a Male-Produced Aggregation Sex Pheromone in *Rosalia batesi*, an Endemic Japanese Longhorn Beetle

**DOI:** 10.3390/insects14110867

**Published:** 2023-11-10

**Authors:** Midori Fukaya, Satoshi Kiriyama, Saki Yagami, Ryûtarô Iwata, Hiroe Yasui, Masahiko Tokoro, Yunfan Zou, Jocelyn G. Millar

**Affiliations:** 1 Department of Forest Science and Resources, College of Bioresource Sciences, Nihon University, Fujisawa 252-0880, Kanagawa, Japan; viridisetviridis@gmail.com (M.F.); lyctus@rk9.so-net.ne.jp (R.I.); 2 Institute for Plant Protection, National Agriculture and Food Research Organization, Tsukuba 305-8666, Ibaraki, Japan; 3 Forestry & Forest Products Research Institute, Tsukuba 305-8687, Ibaraki, Japan; tokoro@affrc.go.jp; 4 Departments of Entomology and Chemistry, University of California, Riverside, CA 92506, USAmillar@ucr.edu (J.G.M.)

**Keywords:** Cerambycidae, Rosaliini, longhorned beetle, male-produced aggregation-sex pheromone, alkylated pyrone

## Abstract

**Simple Summary:**

*Rosalia batesi* Harold (Cerambycidae) is a longhorned beetle endemic to Japan distributed on the mainland from Hokkaido (north) to Kyushu (south). The beautiful appearance of the adults is renowned not only in Japan but worldwide. The larvae feed in the trunks of broad-leaf trees. Females were attracted to males in laboratory bioassays, which suggested that males produce a sex pheromone. We collected volatiles from *R. batesi* females and males and found a single male-specific compound. It was identified as 3,5-dimethyl-6-(1-methylbutyl)-pyran-2-one, which is the same compound identified as the pheromone of its European congener *R. alpina*. This alkylated pyrone is a unique structure among known cerambycid pheromones. In field bioassays carried out with the racemic synthetic pheromone in Japan, both sexes of *R. batesi* were attracted in significant numbers in a ratio of almost 1:1, indicating that the compound is an aggregation-sex pheromone.

**Abstract:**

The longhorned beetle *Rosalia batesi* Harold (Coleoptera; Cerambycidae) is endemic to Japan, where its range extends from Hokkaido to Kyushu. The colorful adults are well-known to entomologists and collectors worldwide. It is a hardwood-boring species with larvae that develop in dead broad-leaf trees. In laboratory bioassays, females were attracted to males, which suggested that males produce a sex pheromone. The congeneric species *R. alpina* is native to Europe, and another congener, *R. funebris,* is distributed in North America. The pheromone components produced by males of these species had been previously identified as two compounds from different biosynthetic pathways. In the present study, volatiles were collected from beetles of both sexes, and the analyses of the resulting extracts revealed a single male-specific compound, which was identified as 3,5-dimethyl-6-(1-methylbutyl)-pyran-2-one; this is the same compound as the pheromone of the European *R. alpina*. This alkylated pyrone structure is, so far, unique among known cerambycid pheromones. In field bioassays with traps baited with the racemic synthetic pheromone, significant numbers of both sexes of *R. batesi* were attracted in an approximately equal ratio, indicating that the compound is an aggregation-sex pheromone rather than a sex pheromone.

## 1. Introduction

*Rosalia batesi* Harold (Coleoptera; Cerambycidae) is endemic to Japan and is distributed on the mainland from Hokkaido to Kyushu. Originally, it was found mainly in the beech belt in mountainous areas, but its distribution has recently expanded to lower elevations, such as hills and plains [1,2,3,4,5,6,7,8,9,10,11,12,13,14]. Host trees of *R. batesi* include species belonging to the genera *Salix*, *Juglans*, *Magnolia*, *Cercidiphyllum*, *Acer*, *Hovenia*, *Styrax*, *Aphananthe, Alnus*, *Aesculus*, and *Zelkova*, all of which are deciduous [15,16,17].

The body size of adults ranges from 16 mm to 30 mm. The sexes are dimorphic, with the length of male antennae being twice as long as the body length, whereas the antennae of females are distinctly shorter. The adults are diurnal and appear from June to August, gathering on sap flows, flowers, and fruits of broad-leaf trees [17]. Females lay eggs on the bark of dead wood, and the developing larvae bore into the heartwood. The larvae may live and develop for years in the dry wood before pupating and emerging as adults. In forest ecosystems, this species plays an important role in the decomposition and recycling of woody biomass [17].

**Figure 1:**
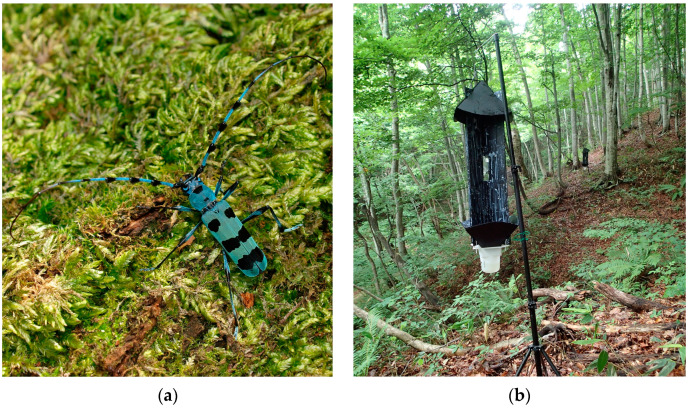
Photographs of (**a**) *Rosalia batesi* (photo by Takehiko Sato); (**b**) black flight-intercept panel traps in field bioassay site (an experimental forest of Nihon Univ., Minakami, Gunma Prefecture, Japan).

*Rosalia* (Rosaliini) is a genus distributed worldwide, and most of the species have spectacular characteristics. Its scientific name, *Rosalia*, means “beautiful maiden” in Latin, which is appropriate for its beauty. The most notable characteristic of Japanese *R. batesi* is its coloration (Figure 1a). The Japanese common name “Ruri-boshi” translates as lapis lazuli color with black dots. Their long blue antennae with black crests are very elegant. In Japan, this species is very popular among people for its beautiful appearance, and it is often depicted on postal stamps and the covers of magazines and books.

Despite its beautiful appearance and image, *R. batesi* can be a pest that attacks wooden building materials, logs, and furniture. As for specific examples of damage, it has attacked the *Alnus japonica* and *Aphananthe aspera* trees of wooden houses [16], and logs of benches and museum exhibits made of processed hardwood [18]. Although this species is not currently designated as a cultural property pest, it is a “drywood pest” because of its ability to feed on the heartwood of trees, and the adaptation of its larvae to dry wood means that it may emerge from processed wooden products [18,19].

The congeneric *Rosalia alpina* is distributed in beech forests in mountainous areas of Europe [1,20,21,22], whereas another congener, *Rosalia funebris,* is found in North American forests [23,24] as the only North American species in its genus. The pheromone components produced by these two species have been identified and, somewhat surprisingly, were entirely different in structure as they were from different biosynthetic pathways. Male *R. alpina* produce 3,5-dimethyl-6-(1-methylbutyl)-pyran-2-one (a polyketide) [25], whereas the major male-specific compound produced by *R. funebris* is (*Z*)-3-decenyl (*E*)-2-hexenoate, likely from fatty acid biosynthetic origins [26]. The alkylated pyrone structure of *R. alpina* pheromone was the first structure of its kind identified as a cerambycid pheromone. Thus, we were interested in identifying the pheromone of the Japanese *R. batesi* to broaden our understanding of the pheromones within the *Rosalia* genus. There is increasing interest in monitoring *Rosalia* beetles in Europe because populations of *R. alpina* are declining, with several countries actively protecting this species [27,28,29]. In contrast, the Japanese *R. batesi* is spreading to lower altitudes, as is *R. alpina*, due to warming global temperatures [2,30].

In our previous studies of possible volatile pheromones for *R. batesi*, among the four combinations tested (male–male, female–male, female–female, and male–female), only the females were attracted to males in laboratory bioassays, which suggested a male-produced sex pheromone [19]. In the mating sequence, encounters generally begin with the female approaching the male. Before the encounter, the males frequently exhibit a “push-up” stance, extending their fore- and mid-legs to raise up the body and bend the abdominal tip. Then, the bifurcate tip is exposed, and it repeatedly opens and closes, which may be related to the emission of volatile pheromones. In the present study, we collected volatiles from both female and male *R. batesi,* analyzed them using coupled gas chromatography–mass spectrometry (GC-MS) and coupled gas chromatography–electroantennographic detection (GC-EAD), and identified the male-specific compound. In field bioassays, the synthesized pheromone proved to be significantly attractive to adults of both sexes, demonstrating that the compound is actually an aggregation-sex pheromone (term after Cardé, 2014 [31]) rather than a sex pheromone per se. The identification of an attractant pheromone should provide a valuable tool for further study of the range, population dynamics, and ecology of this iconic species.

## 2. Materials and Methods

### 2.1. Collection of Insect-Produced Volatiles

No official permits were required for collection of *Rosalia batesi* in Japan. Adult *R. batesi* were hand-collected at Hodaka (36°33′24″ N 137°85′87″ E, altitude 560 m), Nagano Prefecture, Japan on 8 August 2015. Volatile collection was performed at NARO, Tsukuba. Adults were kept individually in plastic cups with a cotton ball soaked with diluted sucrose water. They were provided at 15 °C and 15L9D lighting conditions (lights on 5:00, off 20:00). To collect volatiles, mesh-caged single beetles were held in 100 mL glass beakers covered with aluminum foil and kept for 2 h during the light period at 25 °C [32]. After removal of the beetles, the inside of the beakers was immediately rinsed with 1 mL of *n*-hexane. Three extracts from male beetles and two extracts from females were obtained and kept at −30 °C until analyzed. 

### 2.2. Chemicals

Solvents used for analysis were HPLC grade. A racemic mixture of the regioisomers of the synthetic pyrone was used for verification of the identification of the male-produced compound; its synthesis is described in the supplementary material (Figure S1) [33]. The racemic 3,5-dimethyl-6-(1-methylbutyl)-pyran-2-one used for field bioassays was synthesized as described in Žunič-Kosi et al. (2017) [25]. 

### 2.3. Analysis of Insect-Produced Compounds

Extracts of the male- and female-produced and synthetic pheromones were analyzed at NARO, Tsukuba, on an Agilent 7890A GC system interfaced with a JMS-T100GC Time-of-Flight Mass Spectrometer (GC-TOFMS, JEOL, Tokyo, Japan) in EI mode with 70 eV electron impact ionization at 200 °C. Injections were made in splitless mode at 250 °C with the purge vent closed for 1 min. The GC was fitted with a polar HP-INNOWax column (30 m × 0.25 mm ID × 0.25 μm film; Agilent Technologies, Santa Clara, CA, USA), and the oven temperature was programmed from 50 °C for 1 min, then raised 10 °C per min to 250 °C, then held for 5 min, with helium carrier gas in constant flow mode of 1.1 mL per min. 

Extracts and synthetic compounds were also analyzed on a non-polar DB-1 column (30 m × 0.25 mm ID × 0.25 μm film, Agilent) by using an Agilent 6890N GC linked to a 5973 mass selective detector (Agilent) in EI mode with 70 eV electron impact ionization at 200 °C. Injections were made in splitless mode at 250 °C with the purge vent closed for 1 min. The oven temperature was programmed from 50 °C for 1 min, then raised 10 °C per min to 300 °C, with helium carrier gas in constant flow mode of 1.1 mL per min.

To calculate Kováts indices, a set of linear alkanes from C_12_ to C_28_ was analyzed under the same conditions on each instrument [34].

### 2.4. Field Bioassays of the Synthetic Pheromone

Field bioassays were conducted at an experimental forest of Nihon Univ. (36°48′57″ N 139°02′24″ E, altitude 600–900 m) in Minakami county, Gunma prefecture, Japan from July 16th to August 20th, 2017. Black Teflon^®^ coated flight-intercept panel traps (Alpha Scents Inc., Portland, OR, USA) (panel size: 81 cm × 31 cm; main trap body including hood and collecting funnel: 1 m × 38 cm × 38 cm; collecting cup: 12 cm φ × 20 cm height) were hung along a trail, 1–2 m above the ground, and spaced 15 m apart in a mixed woodland of beech (*Fagus crenata*) and Mizunara oak (*Quercus mongolica var. crispula*) (Figure 1b). The trap collection cups contained ca. 300 mL of propylene glycol (food-grade, ADEKA Corp., Tokyo, Japan) to prevent the escape and decomposition of captured insects.

Traps were baited with the synthetic pheromone candidate (50 mg racemic 3,5-dimethyl-6-(1-methylbutyl)-pyran-2-one in 1 mL isopropanol) and with solvent controls (1 mL isopropanol) (*n* = 4). Lure solutions were deployed in permeable Ziploc plastic sachets made from polyethylene (10 cm × 7 cm, 0.04 mm wall thickness; Unipack C-4, Seinichi, Tokyo, Japan). Each lure was hung over the panel opening (the middle of the panel has an opening) of the trap with wire. Traps were deployed from 16 July to 20 August 2017. Trap catches were counted weekly, with traps being rotated and lures renewed simultaneously. Weekly trap catches with the same lure were recorded for data analyses. Non-target species collected in the traps were also recorded. We referred to the weather data recorded at the Fujiwara Area Weather Station (36°51′8″ N, 139°3′5″ E, altitude 700 m) belonging to the Japan Meteorological Agency (JMA) [URL https://www.jma.go.jp/jma/index.html (accessed on 20 August 2017)], ca. 5 km from the experimental site.

### 2.5. Statistical Analysis

Trap data were analyzed with a general linear model (GLM). The probability distribution of the response variable was a Poisson distribution, and a log function was specified as the link function. Statistical analyses were carried out using R4.1.0 [35].

## 3. Results

### 3.1. Identification of the Male-Produced Volatiles

**Figure 2:**
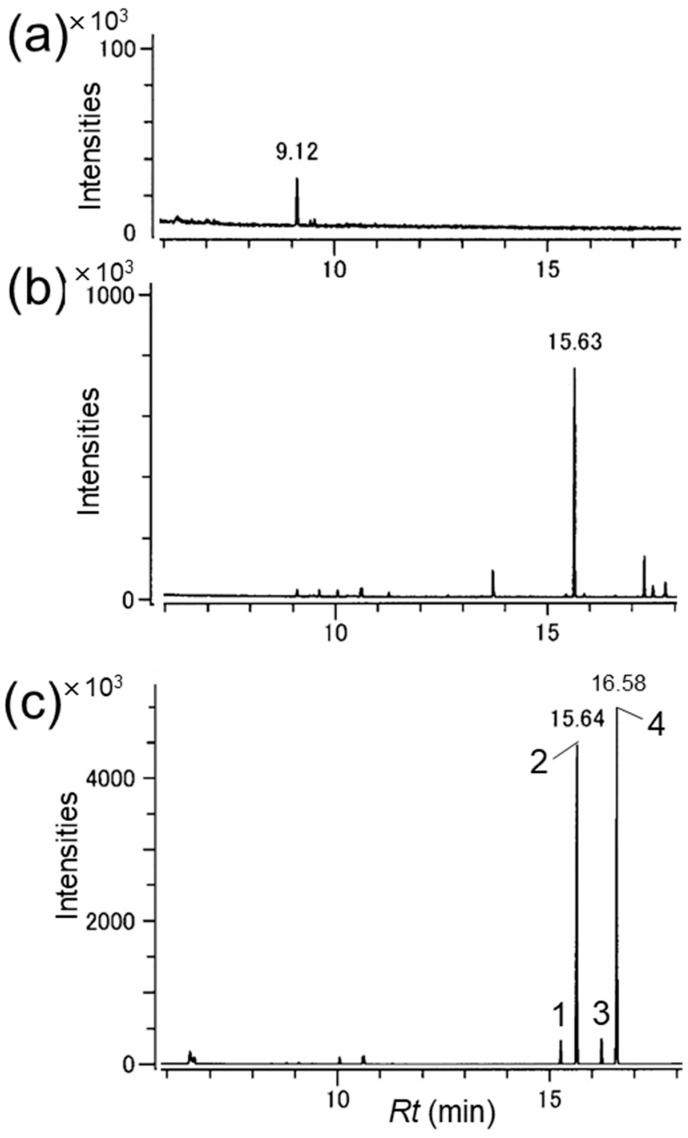
GC profiles of volatiles produced by (**a**) female and (**b**) male *Rosalia batesi*, adsorbed on the surface of a glass beaker; (**c**) synthetic pyrones mixture. HP-INNOWAX column: 30 m × 0.25 mm ID × 0.25 μm film thickness, oven temperature: 50(1)-10-250(5).

**Figure 3:**
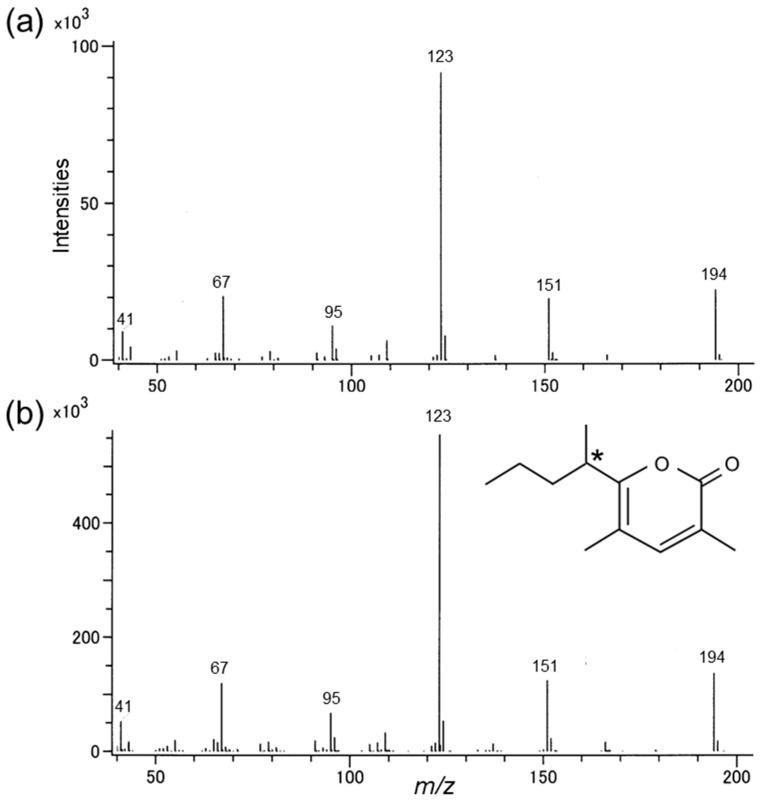
Mass spectra of (**a**) *Rosalia batesi* male-produced compound (15.63 min in Figure 2b) and (**b**) synthetic pyrone (**2**: 15.64 min in Figure 2c) with its chemical structure. *: chiral center.

Extracts of headspace volatiles collected from male and female *R. batesi* were analyzed via GC-TOFMS (Figure 2a,b). A male-specific compound was detected at 15.63 min, and this compound elicited responses from the antennae of females (Figure S1). The mass spectrum of the compound suggested a molecular ion at *m*/*z* 194 (24% of base peak at *m*/*z* 123) and significant ions at *m*/*z* 151 (22%), 123 (base), 95 (12%), and 67 (22%) (Figure 3a). The mass spectrum was similar to that reported for the pyrone compound found in the congeneric species *R. alpina* [25]. Therefore, we compared the compound produced by male *R. batesi* with a synthetic sample of *A. alpina* pheromone and a regioisomer (Figure 2c; for details of synthetic compounds, see Supplementary Material Figure S1). The mass spectrum of the earlier eluting compound **2** (40%) of the synthetic mixture matched that of the male-produced *R. batesi* compound (Figure 3), and the Kováts retention index of the male-produced compound (2119) also matched (synthetic compound **2**: 2120) on the polar HP-INNOWax column. Further analyses via GC-MS determined that the compound had a Kováts index of 1493 (synthetic compound **2**: 1494) on a non-polar DB-1 column. These results confirmed the *R. batesi* male-produced compound as 3,5-dimethyl-6-(1-methylbutyl)-pyran-2-one. The compound was found in amounts estimated at ca. 10 μg/2 h-collection/male but was variable among individuals. The compound was not detected in the extracts prepared from female beetles (Figure 2c).

### 3.2. Field Bioassays

**Figure 4:**
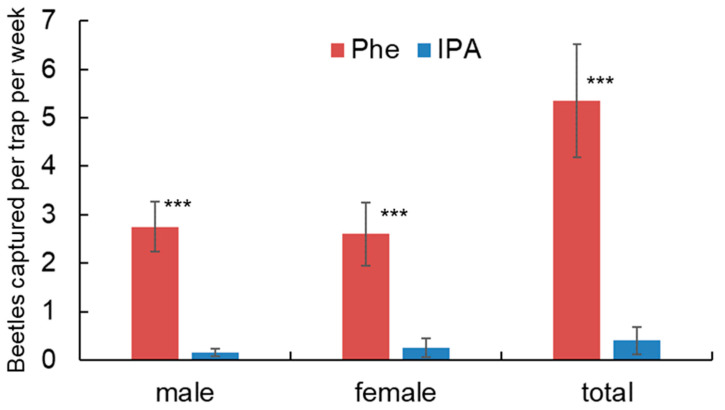
Mean numbers of *Rosalia batesi* captured in traps baited with synthetic pheromone (Phe) and solvent isopropanol (IPA) in a field bioassay in Minakami, Gunma Prefecture, Japan, from 16 July to 20 August 2017. Weekly trap catches with the same lure were recorded for data analyses. Mean ± SEM (bars). *** Significant differences between pheromone treatment and control isopropanol are indicated with asterisks at *p* < 0.0001.

A total of 115 *Rosalia batesi* adults were captured during 5 weeks of field bioassays (107 with pheromone traps and 8 with isopropanol traps). With pheromone lures, 55 males and 52 females were captured. The traps with pheromone lures captured significantly more males (*p* < 0.0001) and females (*p* < 0.0001) than the isopropanol controls, and the total of both sexes was also highly significant (*p* < 0.0001) (Figure 4). The fact that the pyrone attracted both sexes confirmed that this compound acts as an aggregation-sex pheromone [31] rather than a sex pheromone.

**Figure 5:**
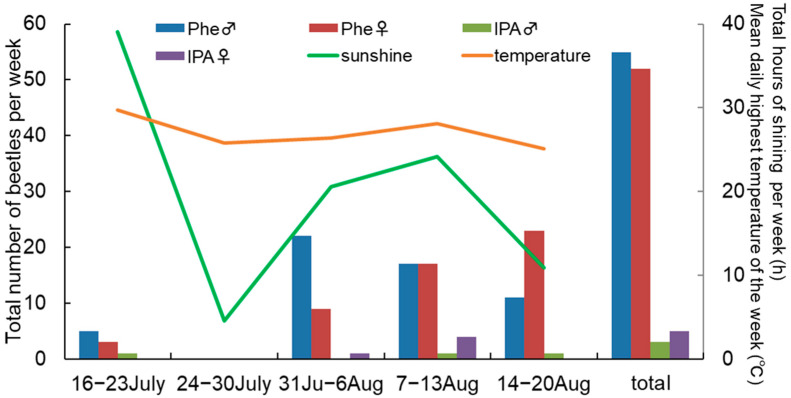
Weekly numbers of *Rosalia batesi* captured in traps in field bioassays. Phe♂/Phe♀; males/females captured with pheromone, IPA♂/IPA♀; males/females captured with control isopropanol. The number of adults trapped in a week shows the total number from 4 traps with the same lure (*n* = 4). The green line shows total hours of sunshine per week, and the orange line shows the mean daily highest temperature of the week at Fujiwara Area Weather Station (JMA), ca. 5 km from the field bioassay site.

The numbers of *R. batesi* captured in pheromone traps differed by week (male: *df* = 1, *p* = 0.012; female: *df* = 1, *p* < 0.001 for a week), with no captures during the second week (Figure 5). The total hours of sunshine per week and the mean daily highest temperature of each week at the assay site are shown in Figure 5. The mean temperature of the second week was not low, but it rained for most of the week. In examining weekly captures of each sex, the ratio of males/females decreased weekly, suggesting that males emerged earlier than females. A total of 68 other cerambycid beetles were caught, including one species of Disteniinae (10 individuals), two species of Prioninae (total of 19 individuals), three species of Lepturinae (total of 4 individuals), three species of Cerambycinae (total of 11 individuals)*,* and 12 species of Lamiinae (total of 24 individuals). However, these captures appeared to be random, with no tendency of pheromone traps to attract particular species (37 in pheromone traps and 31 in isopropanol-baited traps).

## 4. Discussion

The male-specific volatile compound produced by *R. batesi* was identified as 3,5-dimethyl-6-(1-methylbutyl)-pyran-2-one, which is the same compound previously identified as the male-produced pheromone of its European congener *R. alpina.* In field bioassays using the synthetic pheromone, both males and females were attracted to pheromone-baited traps, indicating that the compound is an aggregation-sex pheromone rather than a sex pheromone. The male-specific pheromones of the European *R. alpina* (same pheromone component) and the North American *R. funebris* also work as aggregation-sex pheromones by attracting both sexes [25,26]. To date, almost all volatile pheromones known from species in the subfamily Cerambycinae, Lamiinae, and Spondylidinae are male-produced aggregation-sex pheromones [36,37,38,39,40,41,42,43,44,45], with the single exception being the South African cerambycine *Chorothysa hessei* [46], which has a female-produced sex pheromone. Thus, the pheromone of *R. batesi* is typical for its taxonomic placement.

It is unclear why the pheromones of *R. alpina* and *R. batesi*, which are geographically separated by thousands of miles, are identical, whereas that of *R. funebris* is entirely different. We can only speculate that the ancestral *R. funebris* was introduced into North America over the former land bridge between Alaska and Siberia, and, for some unknown reason, its pheromone changed. This is in distinct contrast to some other cerambycine pheromones, such as 3-hydroxyhexan-2-one, which is so highly conserved that it has been identified as a pheromone, or likely pheromone, for species on all six habitable continents.

In comparing the attraction of the three *Rosalia* species to the synthetic pheromones, the sex ratio of adults captured was male–female 4:44 for *R. funebris* [26], 45:38 for *R. alpina* [25], and 55:52 for *R. batesi* in Japan (this study). The former attracted more females than males, while the latter two species attracted both males and females equally. However, this data should be treated with caution because the sex ratios of these species in nature are not known.

Because these species are not found in high densities, it would make sense to attract both sexes to suitable host plants to increase encounter opportunities by functioning as an aggregation pheromone rather than attracting only the opposite sex as a sex pheromone [31]. In some cerambycid species, attraction to the male-produced aggregation-sex pheromones is increased additively or synergistically by host plant volatiles [47,48,49]. In *R. funebris*, the fact that the adults gather in small aggregations on freshly cut logs and recently dead trees [24] suggests that host plant volatiles play a role in attraction. Thus, it is also possible that host volatiles may act in concert with the pheromone of *R. batesi*. In the case of *R. alpina*, however, there was no evidence that the host plant volatiles (*Z*)-3-hexen-1-ol and ethanol, often used as generic attractants for cerambycids [50], influenced the attraction of adults to pheromone lures [25].

GC-EAD analyses were conducted with the antennae of two males and two females prior to field tests. The antennae of females responded to the male-specific compound in the male extract (Supplementary material Figure S2), but responses from male antennae were inconclusive, in part, because the antennae were difficult to work with, and with a lot of electrical noise in the recordings. 

The pheromone compound identified in the present study has two enantiomeric forms, and the synthetic pheromone used for field bioassays was a racemic mixture. It is possible that the unnatural enantiomer may act as an antagonist, as has been shown for the “non-natural” (*R*)-enantiomer of 2-hydroxyoctan-3-one produced by male *Xylotrechus pyrrhoderus* [43,51]. However, the significant numbers of beetles of both sexes that were attracted suggest that this is not the case here and that the “unnatural” enantiomer of the *R. batesi* pheromone has no effect and may not even be detected. Follow-up studies will be needed to confirm which enantiomer of 3,5-dimethyl-6-(1-methylbutyl)-pyran-2-one is produced by males of both *A. batesi* and *A. alpina*. 

In the laboratory, volatiles from the adults were collected during the day, with some individuals releasing relatively large amounts of pyrone (up to 20 μg/2 h collection). Male-produced pheromones of Cerambycidae have been previously reported to have been produced in large amounts, sometimes more than 30 μg/h [51,52,53]. In the present study, volatiles were collected at 25 °C, whereas the rates of calling individuals were reported to increase when the temperature increased above 26 °C [19]. Thus, if volatile collections were conducted under higher temperatures, the pheromone emission may have increased. Millar and Hanks (2017) [49] pointed out that although the antennae of females are sensitive to nanogram quantities of the pheromones in electroantennogram experiments, males can and do produce much larger amounts [53,54,55]. This highlights the difference between female-produced sex pheromones, which are usually produced in relatively small amounts, and male-produced aggregation-sex pheromones, which mediate the formation of the conspecific aggregations commonly observed [56]. There is also evidence that calling males stimulate other males to call in a type of “chorusing” behavior [41]. These points suggest that aggregation-sex pheromones are also involved in sexual selection, with males competing to advertise their fitness to females via the production of large amounts of pheromones. 

The zero trap captures in the second week of the five-week field bioassays were likely due to bad weather during that week. When the temperature rises above a threshold level, the beetle’s activity increases [17], and calling behavior is typically observed when the body surface temperature of males is higher than 26 °C, which is associated with light intensity [19]. During the second week of the field bioassay, the weather was rainy, so the lack of catch in any traps may have been due to the body surface temperature not rising enough in addition to the rain possibly suppressing flight. The capture of males also decreased from weeks 3 to 5, suggesting that females emerge later than males in the field. 

The availability of a pheromone that is attractive to both sexes should provide a valuable tool for further studies of the range, population dynamics, and ecology of this iconic species. Prior to this study, the only way to find *R. batesi* was by visually searching, and the only information available on its distribution was from reports of sightings. However, now that aggregation pheromones have been identified, pheromone traps may be used to readily monitor the distribution and population densities of this species as it continues to expand its range. Because the pheromone is specific to this species in Japan, by-catch of nontarget species will be minimized. Pheromone-baited traps can be powerful tools for monitoring insect species, especially at low population densities, as is often the case with endangered and newly invasive species [38,49,57,58]. Monitoring endangered species and invasive species differ in concept and protocols [59,60]. Various factors must be considered in developing pheromone lures for practical use, such as the cost of synthesis of the pheromone and the effective lifetime of lures. In particular, lures for cerambycid aggregation-sex pheromones need to release relatively large amounts of pheromone (~1 mg/d) to mimic the natural release rates [49]. Thus, studies to determine the best dose and the optimal release device would be beneficial for the development of effective and cost-effective lures for *R. batesi*.

## 5. Conclusions

The male-specific compound produced by Japanese *R. batesi* was identified as a pyrone compound identical to that emitted by the European *R. alpina* but different from the pheromone of the North American *R. funebris*. To date, the alkylated pyrone is a unique structure among known cerambycid pheromones [25,49]. The geographic distributions of the two former *Rosalia* species are separated by thousands of kilometers, so it is noteworthy that the pheromone has been conserved. The significant catches of both sexes of *R. batesi* in pheromone-baited traps indicate that such traps may be useful for further studies of the distribution and ecology of *R. batesi*.

## Data Availability

Data are available upon request.

## Supplementary Materials

The following supporting information can be downloaded at: https://www.mdpi.com/article/10.3390/insects14110867/s1, Figure S1: Rapid synthesis of target pyrone as a mixture of regioisomers; Figure S2: Analysis of extracts by coupled gas chromatography-electroantennogram detection (GC-EAD).

## References

[1] Takakuwa M., Takakuwa M. (1998). World’s *Rosalia*. The Rosalia World.

[2] Takakuwa M. (2000). Distributional expansion of a cerambycine longicorn beetle, *R. batesi*, in southern Fossa Magna area, central Japan, with special references to its surroundings. Gekkan-Mushi.

[3] Kanno Y., Ito Y. (2000). Record of *Rosalia batesi* (Coleoptera: Cerambycidae) in Yokohama-city. Gekkan-Mushi.

[4] Hinoki T. (2001). Records of *Rosalia batesi* (Coleoptera: Cerambycidae) from Tama-city, Tokyo. Gekkan-Mushi.

[5] Kanno Y. (2001). Additional records of *Rosalia batesi* (Coleoptera: Cerambycidae) in Yokohama-city. Gekkan-Mushi.

[6] Kawada K. (2001). Records of *Rosalia batesi* (Coleoptera: Cerambycidae) in Kawasaki-city in Kanagawa Prefecture. Gekkan-Mushi.

[7] Saito K. (2001). Additional records of *Rosalia batesi* (Coleoptera: Cerambycidae) and *Parnassius citrinarius* (Lepidoptera: Papilionidae) in expanding distribution area. Gekkan-Mushi.

[8] Ichihashi H. (2005). Distribution of *Rosalia batesi* (Coleoptera: Cerambycidae) in Sagamihara-city in Kanagawa Prefecture. Gekkan-Mushi.

[9] Miyauchi H. (2007). Record of *Rosalia batesi* (Coleoptera: Cerambycidae) in Sodegaura-city in Chiba Prefecture. Gekkan-Mushi.

[10] Makihara H., Nakamura N. (2009). Records of *Rosalia batesi* (Coleoptera: Cerambycidae) in Shimodate-city in Ibaraki Prefecture. Gekkan-Mushi.

[11] Dejima T. (2009). Record of *Rosalia batesi* (Coleoptera: Cerambycidae) from Kagawa Prefecture. Gekkan-Mushi.

[12] Suda H. (2009). Record of *Rosalia batesi* (Coleoptera: Cerambycidae) in Kawagoe-city in Saitama Prefecture. Gekkan-Mushi.

[13] Takahashi K. (2010). Records of *Rosalia batesi* (Coleoptera: Cerambycidae) in Hiratsuka-city in Kanagawa Prefecture. Kanagawa Pref. Plant Prot..

[14] Hirano M., Higashijima K., Koga Y., Ochi T. (2017). Distributional expansion of *Rosalia batesi* in central Kinki district, Japan. Gekkan-Mushi.

[15] Kojima K., Nakamura S. (1986). Food plants of cerambycid beetles (Cerambycidae, Coleoptera) in Japan. Hiba Soc. Nat. Hist. Shobara.

[16] Yamaguchi K. (1998). A wooden house damaged by *Rosalia batesi* Harold (Coleoptera: Cerambycidae). House Househ. Insect Pests.

[17] Iwata R., Aoki M., Nozaki T., Yamaguchi M. (1998). Some notes on the biology of a hardwood-log-boring beetle, *Rosalia batesi* Harold (Coleoptera: Cerambycidae), with special reference to its occurrences in a building and a suburban lumberyard. Jpn. J. Environ. Entomol. Zool..

[18] Kiriyama S., Iwata R. (2013). Two cases of indoor occurrences of *Rosalia batesi* Harold (Coleoptera: Carambycidae) emerged from lumbers at Shonan Campus, Nihon University, Fujisawa, Japan. Urban Pest Manag..

[19] Kiriyama S., Iwata R., Fukaya M., Hoshino Y., Yamanaka Y. (2018). Mating behavior of *Rosalia batesi* (Coleoptera: Cerambycidae) is mediated by male-produced sex pheromones. Insects.

[20] Sama G., Sama G. (2002). Subfamily Cerambicinae Latreille, 1802. Atlas of the Cerambycidae of Europe and the Mediterranean Area.

[21] Lachat T., Ecker K., Duelli P., Wermelinger B. (2013). Population trends of *Rosalia alpina* (L.) in Switzerland: A lasting turnaround?. J. Insect Conserv..

[22] Michalcewicz J., Ciach M. (2015). Current distribution of the Rosalia longicorn *Rosalia alpina* (Linnaeus, 1758) (Coleoptera: Cerambycidae) in Poland. Pol. J. Entomol..

[23] Linsley E.G. (1964). The Cerambycidae of North America: Part V. Taxonomy and classification of the subfamily Cerambycinae, tribes Callichromini through Ancylocerini. Univ. Calif. Publ. Entomol..

[24] Linsley E.G. (1995). The banded alder beetle in natural and urban environments (Coleoptera: Cerambycidae). Pan-Pac. Entomol..

[25] Žunič Kosi A., Zou Y., Hoskovec M., Vrezec A., Stritih N., Millar J.G. (2017). Novel, male-produced aggregation pheromone of the cerambycid beetle *Rosalia alpina*, a priority species of European conservation concern. PLoS ONE.

[26] Ray A.M., Millar J.G., McElfresh J.S., Swift I.P., Barbour J.D., Hanks L.M. (2009). Male-produced aggregation pheromone of the cerambycid beetle *Rosalia funebris*. J. Chem. Ecol..

[27] IUCN (2023). The IUCN Red List of Threatened Species. Version 2022.2. www.iucnredlist.org.

[28] Komonen A., Jonsell M., Ranius T. (2008). Red-listing saproxylic beetles in Fennoscandia: Current status and future perspectives. Endanger. Species Res..

[29] Drag L., Hauck D., Pokluda P., Zimmermann K., Cizek L. (2011). Demography and dispersal ability of a threatened saproxylic beetle: A mark-recapture study of the Rosalia longicorn (*Rosalia alpina*). PLoS ONE.

[30] Cizek L., Schlaghamerský J., Bořucký J., Hauck D., Helešic J. (2009). Range expansion of an endangered beetle: Alpine longhorn *Rosalia alpina* (Coleoptera: Cerambycidae) spreads to the lowlands of Central Europe. Entomol. Fenn..

[31] Cardé R.T. (2014). Defining attraction and aggregation pheromones: Teleological versus functional perspective. J. Chem. Ecol..

[32] Yasui H., Wakamura S., Arakaki N., Yasuda T., Akino T., Fukaya M. (2007). Collection and quantification of airborne pheromone from individual females of the black chafer *Holotrichia loochooana loochooana* (Coleoptera: Scarabaeidae): Heterogeneity of feral females in respect to pheromone release. Appl. Entomol. Zool..

[33] Itoh M., Shimizu M., Hirano K., Satoh T., Miura M. (2013). Rhodium-catalyzed decarboxylative and dehydrogenative coupling of maleic acids with alkynes and alkenes. J. Org. Chem..

[34] Kovát E. (1965). Gas chromatographic characterization of organic substances in the retention index system. Adv. Chromatogr..

[35] R Core Team (2021). R: A Language and Environment for Statistical Computing.

[36] Allison J.D., Borden J.H., Seybold S.J. (2004). A review of the chemical ecology of the Cerambycidae (Coleoptera). Chemoecology.

[37] Fettköther R., Dettner K., Schröder F., Meyer H., Francke W., Noldt U. (1995). The male pheromone of the old house borer *Hylotrupes bajulus* (L.) (Coleoptera: Cerambycidae): Identification and female response. Experientia.

[38] Hanks L.M., Millar J.G. (2016). Sex and aggregation-sex pheromones of cerambycid beetles: Basic science and practical applications. J. Chem. Ecol..

[39] Hansen L., Xu T., Wickham J., Chen Y., Hao D., Hanks L.M., Millar J.G., Teale S.A. (2015). Identification of a male-produced pheromone component of the citrus longhorned beetle, *Anoplophora chinensis*. PLoS ONE.

[40] Iwabuchi K., Takahashi J., Nakagawa Y., Sakai T. (1986). Behavioral responses of female grape borer to synthetic male sex pheromone components. Appl. Entomol. Zool..

[41] Lemay M.A., Silk P.J., Sweeney J. (2010). Calling behavior of *Tetropium fuscum* (Coleoptera: Cerambycidae: Spondylidinae). Can. Entomol..

[42] Noldt U., Fettköther R., Dettner K. (1995). Structure of the sex pheromone-producing prothoracic glands of the male old house borer, *Hylotrupes bajulus* (L.) (Coleoptera: Cerambycidae). Int. J. Insect Morphol. Embryol..

[43] Sakai T., Nakagawa Y., Takahashi J., Iwabuchi K., Ishii K. (1984). Isolation and identification of the male sex pheromone of the grape borer *Xylotrechus pyrrhoderus* Bates (Coleoptera: Cerambycidae). Chem. Lett..

[44] Silk P.J., Sweeney J., Wu J., Price J., Gutowski J.M., Kettela E.G. (2007). Evidence for a male-produced pheromone in *Tetropium fuscum* (F.) and *Tetropium cinnamopterum* (Kirby) (Coleoptera: Cerambycidae). Naturwissenschaften.

[45] Teale S.A., Wickham J.D., Zhang F., Su J., Chen Y., Xiao W., Hanks L.M., Millar J.G. (2011). Male-produced aggregation pheromone of *Monochamus alternatus* (Coleoptera: Cerambycidae), a major vector of pine wood nematode. J. Econ. Entomol..

[46] Cohen C., Liltved W.R., Colville J.F., Shuttleworth A., Weissflog J., Svatoš A., Bytebier B., Johnson S.D. (2021). Sexual deception of a beetle pollinator through floral mimicry. Curr. Biol..

[47] Ginzel M.D., Hanks L.M. (2005). Role of host plant volatiles in mate location for three species of longhorned beetles. J. Chem. Ecol..

[48] Hanks L.M. (1999). Influence of the larval host plant on reproductive strategies of cerambycid beetles. Annu. Rev. Entomol..

[49] Millar J.G., Hanks L.M., Wang Q. (2017). Chemical ecology of Cerambycids. Cerambycidae of the World: Biology and Pest Management.

[50] Miller D.R. (2006). Ethanol and (−)-α-pinene: Attractant kairomones for some large wood-boring beetles in southeastern USA. J. Chem. Ecol..

[51] Iwabuchi K. (1986). Mating behavior of *Xylotrechus pyrrhoderus* Bates. III. Pheromone secretion by male. App. Entomol. Zool..

[52] Iwabuchi K., Takahashi J., Sakai T. (1987). Occurrence of 2,3-octanediol and 2-hydroxy-3-octanone, possible male sex pheromone in *Xylotrechus chinensis*. Appl. Entomol. Zool..

[53] Hall D.R., Cork A., Phythian S.J., Chittamuru S.B., Jayarama K., Venkatesha M.G., Sreedharan K., Vinod Kumar P.K., Seetharama H.G., Naidu R. (2006). Identification of components of male-produced pheromone of coffee white stem borer, *Xylotrechus quadripes*. J. Chem. Ecol..

[54] Pajares J.A., Álvarez G., Ibeas F., Gallego D., Hall D.R., Farman D.I. (2010). Identification and field activity of a male-produced aggregation pheromone in the pine sawyer beetle, *Monochamus galloprovincialis*. J. Chem. Ecol..

[55] Zhang A., Oliver J.E., Aldrich J.R., Wang B., Mastro V.C. (2002). Stimulatory beetle volatiles for the Asian longhorned beetle, *Anoplophora glabripennis* (Motschulsky). Z. Naturforsch..

[56] Hanks L.M., Wang Q., Wang Q. (2017). Reproductive biology of Cerambycids. Cerambycidae of the World: Biology and Pest Management.

[57] Larsson M.C. (2016). Pheromones and other semiochemicals for monitoring rare and endangered species. J. Chem. Ecol..

[58] Musa N., Andersson K., Burman J., Andersson F., Hedenström E., Jansson N., Paltto H., Westerberg L., Winde I., Larsson M.C. (2013). Using sex pheromone and a multi-scale approach to predict the distribution of a rare saproxylic beetle. PLoS ONE.

[59] Andersson K., Bergman K.O., Andersson F., Hedenström E., Jansson N., Burman J., Winde I., Larsson M.C., Milberg P. (2014). High-accuracy sampling of saproxylic diversity indicators at regional scales with pheromones: The case of *Elater ferrugineus* (Coleoptera, Elateridae). Biol. Conserv..

[60] Bosso L., Rebelob H., Garonnac A.P., Russo D. (2013). Modelling geographic distribution and detecting conservation gaps in Italy for the threatened beetle *Rosalia alpina*. J. Nat. Conserv..

