# Supplementary material for: Identification of a Male-Produced Aggregation Sex Pheromone in Rosalia batesi, an Endemic Japanese Longhorn Beetle
Source: Insects. 2023 Nov 10;14(11):867. doi: 10.3390/insects14110867 (PMC10671707; doi:10.3390/insects14110867)
Supplement: Supplementary file 1 [file insects-14-00867-s001.zip › insects-2698090-supplementary.pdf]

Supplementary Materials

Figure S1

Rapid synthesis of target pyrone as a mixture of regioisomers.

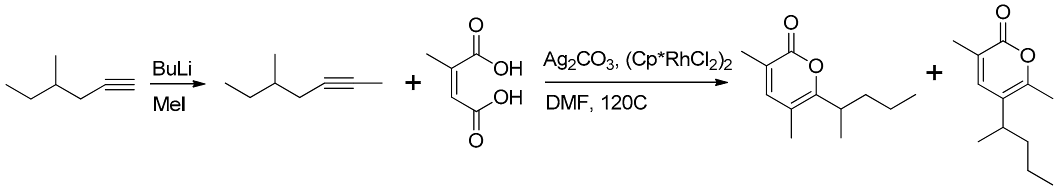

Synthesis of pheromone compounds was conducted at UC Riverside. A short synthesis of the target pyrone as a mixture of regioisomers was developed based on a rhodium-catalyzed decarboxylative coupling of citraconic acid and 4-methyl-2-heptyne [33] to provide a standard to verify the structure, as described below. A second regiospecific synthesis was then developed, as described in Žunič-Kosi et al. (2017) [25], to provide gram quantities of material for field bioassays.

A solution of 3-methyl-1-hexyne (4.8 g, 50 mmol) and ~50 mg triphenylmethane indicator in 80 ml dry THF under argon was cooled to ~−10 °C, and BuLi (25 mL of a 2.2 M solution in hexanes) was added dropwise, producing a bright red-orange solution. Methyl iodide (8.52 g, 60 mmol) was then added dropwise over 20 min, and the resulting mixture was warmed to room temp overnight, stirred at room temp for 24 h, then warmed to 42 °C for 2 h. After cooling to room temp, the mixture was quenched with ice cold saturated NH_4Cl solution and extracted with pentane. The organic layer was washed with brine, dried over anhydrous Na_2SO_4, and the bulk of the solvent was distilled off at atmospheric pressure with a jacketed Vigreux column. The residue was then fractionally distilled at 120 mm Hg, taking a forerun with bp < 50 °C, then distilling the bulk over at 60–62 °C at 120 mm Hg, yielding 3.89 g of 4-methyl-2-heptyne as a colorless volatile liquid.

A dry 500 mL 3-neck flask was charged with citraconic acid (3.9 g, 30 mmol), Ag_2CO_3 (8.28 g, 30 mmol), pentamethylcyclopentadienyl rhodium dichloride dimer ((Cp*RhCl$_2$)$_2$, 0.64 mmol), and flushed thoroughly with Ar. Dry DMF (40 mL) was then added, followed by 4-methyl-2-heptyne (3.3 g, 30 mmol) and an additional 60 mL DMF. The mixture was then heated under Ar at 120 °C for 3 h, during which time the mixture turned from brown to black, with CO_2 outgassing, then yellowish to rad-brown, darkening to black with precipitation. The mixture was cooled to room temp, poured into 1 L icewater, and extracted 3 times with pentane. The combined pentane layers were filtered, giving a clear orange solution, which was washed with water and brine, dried over anhydrous Na_2SO_4, and concentrated to 6.23 g orange oil. The oil was purified by flash chromatography on silica gel, eluting with 7% acetone in hexane, then Kugelrohr distilled (bp ~90 °C, 0.1 mm Hg), yielding 4.53 g of a mixture of 4 pyrones (4:40:4:52 on DB-5 GC column) as a viscous yellow oil. First isomer (4%),

EIMS, 70 eV (*m*/*z*, abundance): 194 (M^+,32), 165 (10), 137 (100), 123 (12), 109 (16), 95 (13), 67 (19), 43 (7), 41 (9). Second isomer (40%): 194 (M^+, 52), 166 (3), 151 (41), 123 (100), 109 (10), 95 (17), 67 (29), 43 (8), 41 (13).Third isomer (4%) 194 (M^+, 29), 165 (100), 137 (23), 123 (18), 109 (6), 95 (10), 77 (6), 67 (3), 65 (3), 43 (42), 41 (3). Fourth isomer: 194 (M^+, 53), 179 (5), 166 (2), 137 (14), 123 (57), 109 (14), 95 (12), 79 (10), 77 (10), 67 (3), 43 (46), 41 (6). The 40% isomer matched the retention time and mass spectrum of the insect-produced compound, and those of the single isomer that was subsequently synthesized by the regioselective route described in Žunič Kosi et al. (2017) [25].

Figure S2

Analysis of extracts by coupled gas chromatography-electroantennogram detection (GC-EAD).

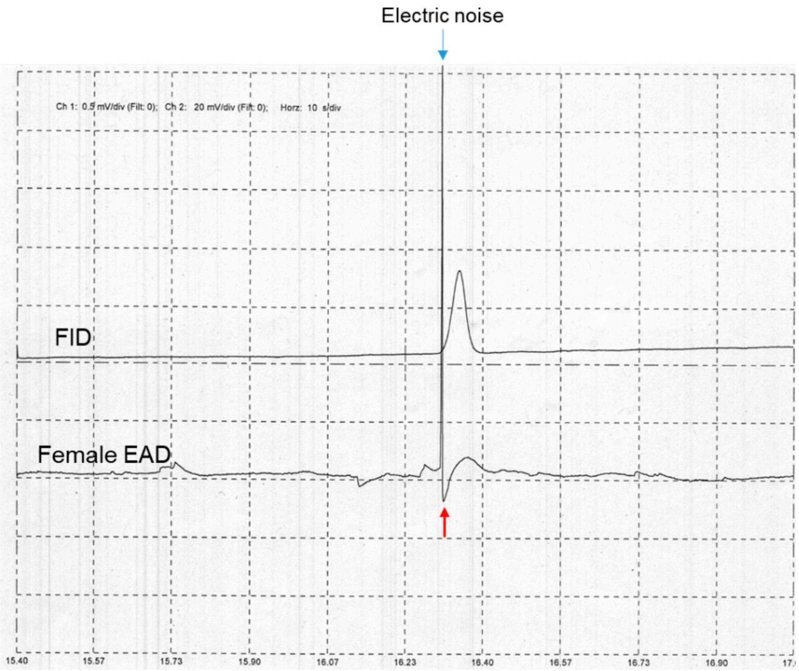

The top trace shows the GC detector response to the natural compound emitted by male *Rosalia batesi*; and the bottom trace shows the response from an antenna of a female *R. batesi*. The red arrow indicates the corresponding EAG response to the main natural compound and the blue arrow indicates electric noise.

The extracts of beetle volatiles were analyzed by GC-EAD using a Hewlett-Packard 5890 series II GC fitted with a HP-INNOWax column (30 m × 0.32 mm ID × 0.25 µm film thickness, Agilent Technologies, Santa Clara, CA, USA) at FFPRI, Tsukuba. The injector and detector temperatures were 250 °C, and injections were made in splitless mode. The oven was programmed from 50 °C for 1 min, then 10 °C per min to 250 °C, using helium carrier gas at

a linear 50 cm/s velocity. The FID detector temperature was 250 °C. Helium makeup gas (10 mL per min) was added to the column effluent via a stainless steel T-union, after which the flow was split equally between the GC's flame ionization detector (FID) and the EAD with a press-fit Y splitter (Agilent Technologies). The GC effluent for EAD was directed to a glass transfer tube (15 mm ID) mounted on the GC and was mixed with humidified air (300 mL per min, 20 °C), and then passed over the antennal preparation. An antenna including the basal segment was gently removed from a live beetle with scissors and forceps, and was mounted on a Syntech EAG probe (Syntech, Kirchzarten, Germany), making connections with electrode gel (Spectra® 360, Parker Lab. Inc., Fairfield, NJ, USA). EAG and FID signals were fed into a computer through an analogue-to-digital conversion board (IDAC-232, Syntech), and the signals were displayed and analyzed with Syntech GC–EAD software. In total, analyses were replicated with antennae from two females and two males, with each antennal preparation being reused for 2-3 analyses. Unfortunately, the antennal preparations displayed a lot of electrical noise, and clear EAG responses were not confirmed with male antennae.
